# A systematic review and thematic synthesis to identify factors that influence pharmacists' involvement in asthma care services: An identity crisis

**DOI:** 10.1016/j.rcsop.2021.100051

**Published:** 2021-07-27

**Authors:** Amnah Taqi, Gill Rowlands, Adam Pattison Rathbone

**Affiliations:** a School of Pharmacy, Faculty of Medical Sciences, Newcastle University, United Kingdom; b Population and Health Sciences Institute, Faculty of Medical Sciences, Newcastle University, United Kingdom

**Keywords:** Asthma, Pharma, Qualitative, Experience, Attitude, Belief, Behaviour

## Abstract

**Background:**

Asthma is a common chronic disease worldwide affecting an estimated 300 million people. Pharmacists can play key roles to support optimal health outcomes for patients with asthma. Goffman's Dramaturgical Theory was used in this review to critically examine the literature describing the role of pharmacists in asthma services.

**Objectives:**

The aim of this review is to identify factors that influence the role of pharmacists in asthma care services.

**Methods:**

A systematic literature search was conducted of seven electronic databases including: CINAHL, Midline (Ovid), PubMed, Scopus, Web of science, Embase and PsycInfo.). The search was not restricted by language or date of publication. Studies were screened according to inclusion criteria which included much relate to pharmacists, asthma services and include qualitative findings. Data was extracted and thematically synthesised to create demographic, descriptive and analytical findings.

**Results:**

Eighteen studies were included. The majority of studies were conducted in high income countries, with most of the studies conducted in Australia (*n* = 10). Semi-structured interview was used as a method for data collection in most studies (*n* = 11). Evidence indicated pharmacists engaged in asthma services positively and wanted to expand their roles in patient care. However, literature reported patients' attitudes and health-system factors such as remuneration, as well as inter-professional collaboration and expected low levels of knowledge and skills of pharmacists were barriers to implementation of pharmacy-led asthma care. Analytical findings suggest that pharmacists' involvement in asthma care services were influenced by patients' and healthcare professionals' expectations which were juxtaposed with pharmacists' own self-perceived identity.

**Conclusions:**

This review demonstrates pharmacists self-identified as being capable and equipped with appropriate knowledge and skills, however the expectations of patients and other healthcare professionals prohibited their involvement in delivering asthma care services.

## 1 Introduction

Asthma is a respiratory disease caused by chronic airway inflammation in the lungs that constricts the airway and increases mucus production.^1^ It is one of the most common chronic diseases worldwide, affecting an estimated 300 million people.^1^ Asthma is characterized by recurrent respiratory symptoms, such as wheezing, breathlessness, chest tightness and cough that may have an adverse impact on the quality of life of patients and their families.^1^ Delivering better services for people with asthma requires collaborative efforts between healthcare professionals in multidisciplinary teams, including pharmacists. General practitioners (GPs) play a central role in the management of asthma that involves assessment, diagnosis, prescription of regular medications, education and provision of written action plans, while nurses have a role in practical guidance on taking medicines, how to remember to take the medicine, how to recognize and to remember how to increase medication during exacerbation and inhaling technique. Over the last two decades, there has been a paradigm shift in pharmaceutical services with pharmacists expanding their roles from dispensing to the provision of a variety of clinical services for different diseases.^2^ Pharmacists are uniquely positioned to support optimal health outcomes for patients with asthma due to their expertise in medications used to treat the disease, their frequent contact with patients and their accessibility.^3^ Evidence showed that pharmacists can play key roles in asthma and pharmacists' interventions can result in improved asthma control and quality of life.^4^^,^^5^ These interventions include providing patient education regarding self-management, training on inhalation technique, addressing patients' concerns of medications' side effects and disease monitoring.^3^ However, little is known about what influence pharmacists' experiences and behaviours when providing asthma care services.

In this review, Goffman's Dramaturgical Theory was used to critically examine the literature describing the role of pharmacists in asthma services. Goffman described people as actors on a social stage who actively create an impression of themselves through the performance of behaviours.^6^^,^^7^ Goffman believed that individuals use impression management to present themselves to others as they would wish to be perceived. This conceptual framework is linked to a theatrical play where our daily life is the ‘stage’, the individual is the ‘social actor’ and the people around the individual are the ‘audience’. According to Goffman's theory, there are ‘on stage’ behaviours, where actors are on stage performing a role, using their perception of the audience and the audiences' expectations for the role they should play to influence their behaviour. Also, there are ‘backstage’ behaviours, where actors can relax and behave out of ‘character’ to the role or identity that they play when they are in front of others.

Although pharmacists are highly accessible professionals and they can be contributors to improve asthma care, there have been limited opportunities to use pharmacists' skills specifically for the medication management of patients with asthma.^8^ Implementation science theories suggest that the first step in the development of most health services is to study the perspectives of practitioners and patients who might be involved in the services being implemented.^9^ If such interventions or services are to become part of practice, adequate uptake of the intervention by patients and health professionals in the ‘real world’ is needed. For example, it is important to explore the willingness of pharmacists to expand their role in providing asthma care and what factors influence their involvement and behaviours providing asthma care services. This information could lead to the development of service models in which pharmacists provide asthma care away from their conventional role of dispensing and medication supply.^8^ The aim of this review is to explore the evidence of pharmacists' involvement in asthma care services and to critically examine the role of the pharmacist in asthma care services.

## 2 Methods

Qualitative research provides a detailed picture of participants' feelings, beliefs, attitudes and experiences and interprets the meanings of their actions.^10^ Additionally, qualitative studies deliver a contextualised feeling of the world that can inform the development of new models to improve care.^11^ A qualitative review allows a researcher to describe, interpret and synthesise a given topic, leading to the identification of common and emergent themes in extant literature.^12^ Although a quantitative systematic review and meta-analysis may capture the role of pharmacists in asthma care services (e.g. how many are involved?) in a generalisable way, as an exploratory study, the qualitative approach to systematic review and thematic synthesis was appropriate as a starting point to answer foundational questions (e.g. how are they involved?). This will allow future work to quantify the roles of pharmacists in asthma care services based on evidence-based understanding.

### 2.1 Search strategy and key words

**Fig. 1:**
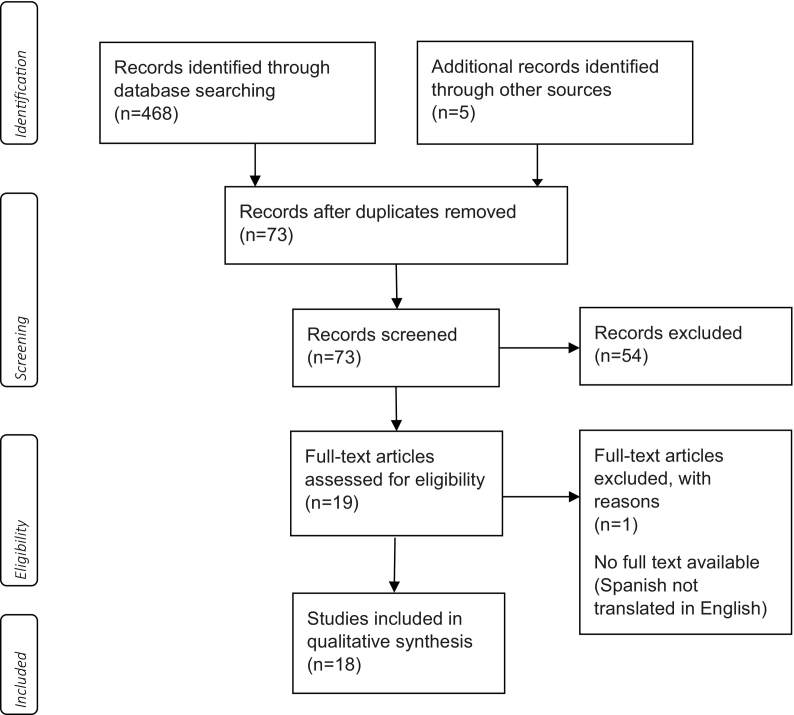
PRISMA diagram.

A systematic literature search was conducted of seven electronic databases including: CINAHL, Midline(ovid), PubMed, Scopus, Web of science, Embase and PsycInfo. The search strategy involved searching each database individually using the appropriate term in each one. Keywords searched included “asthma”, “pharma*”, “qualitative”, “experience*”, “attitude*”, “perception*”, “perspective*”, “belief*”, “view*”, “behaviour*” and “opinion*”. Keywords were combing using AND/OR (see Table 1 below). The search was not restricted by language or date of publication. The search strategy included searching the reference lists of primary articles for additional relevant papers. The literature search was conducted in February 2021. A.T was involved in the search and inclusion/exclusion processes and then all the processes were reviewed by other listed authors. After screening, 18 relevant studies were included in the review (see PRISMA Diagram in Fig. 1). This study has been registered with PROSPERO (registration number is: CRD42021247456).

**Table 1:** Databases and search terms.

Database	Keywords and search terms
PubMed	Asthma AND pharma* AND qualitative AND (experience* OR attitude* OR perception* OR perspective* OR belief* OR view* OR behaviour* OR opinion*)
Scopus	(TITLE-ABS-KEY (asthma) AND TITLE-ABS-KEY (pharma*) AND TITLE-ABS-KEY (qualitative) AND TITLE-ABS-KEY (experience* OR attitude* OR perception* OR perspective* OR belief* OR view* OR behaviour* OR opinion*))
CINAHL	AB asthma AND AB pharma* AND AB qualitative AND AB (experience* OR attitude* OR perception* OR perspective* OR belief* OR view* OR behaviour* OR opinion*)
Medline (ovid), Embase and PsycInfo	Asthma AND pharma* AND qualitative AND (experience* OR attitude* OR perception* OR perspective* OR belief* OR view* OR behaviour* OR opinion*)
Web of science	(AB = (asthma AND pharma* AND qualitative AND (experience* OR attitude* OR perception* OR perspective* OR belief* OR view* OR behaviour* OR opinion*))) *AND* DOCUMENT TYPES: (Article) Indexes = SCI-EXPANDED, SSCI, A&HCI, CPCI-S, CPCI-SSH, ESCI Timespan = All years

### 2.2 Inclusion criteria

The following inclusion criteria was used:

1. Studies reporting qualitative findings.
  a. Including studies using mixed or quantitative methods that had qualitative elements.
2. Studies focusing on asthma
  a. Including studies that focused on asthma in addition to other diseases.
3. Studies focusing on pharmacists' opinions, attitudes, beliefs and experiences.
  a. Including studies that involved other healthcare professionals if pharmacists were also involved.
  b. Including studies that involved patients if pharmacists were also involved.
4. Studies available in English or Arabic.

### 2.3 Exclusion criteria

The following exclusion criteria was used:

1. Studies that did not report qualitative findings.
2. Studies not focusing on asthma.
3. Studies that did not include pharmacists.
4. Studies focusing solely on patient experience.
5. Studies not available in English or Arabic.

### 2.4 Quality assessment and assessment of bias

One author used the CASP Qualitative Research Checklist Tool. Appraisal was quality checked by the remaining authors (APR/GR) to ensure consistency of assessment. Variation in the quality of studies was recognised across the studies (See Table 2). The quality of the studies was categorised into weak, medium and strong evidence according to quality rating score (CASP). As shown in Table 2, quality rating score (1−2−3) indicates weak evidence, (4–5-6) indicates medium evidence and (7–8-9) indicates strong evidence.

**Table 2:** Summary of included literature.

Author /Date /country	Title of article	Setting	Study design/ Data collection	Participants	Summary	Strengths	Limitations	CASP tool
15 Australia	It's a powerful message: a qualitative study of Australian healthcare professionals' perceptions of asthma through the medium of drawings.	Primary and tertiary health care centres	A qualitative exploratory study in which healthcare professionals (HCPs) expressed their perspectives of asthma in a drawing followed by a review of drawings made by asthmatic patients	A sample of three GPs, two respiratory physicians, 10 Pharmacists, five nurses and three physiotherapists	Participants' perspectives were largely biomedically centred, illustrating physiological and clinical aspects of asthma. The patients' drawings encouraged the participants to revisit their personal expectations of patients' illness experience; triggered acknowledgement and empathy regarding the patient experience; and supported clinical reflexivity. Pharmacists perceived themselves to be competent in the medical aspects of the condition, but they felt less confident about their understanding of patients' real experiences. Pharmacists stated that they frequently received neutral responses when they asked patients about their asthma. Time limitation was mentioned as a major barrier to investigate patient's experience and act on their empathy.	-First study to investigate healthcare professionals' perspectives with patients' lived experience of asthma through the medium of drawings. -The perspectives of patients with asthma were included in the study through their drawings. -Both primary and tertiary HCPs were included.	-There were fewer medical and more allied HCPs.	9
16 Australia	Perceived feasibility of a community pharmacy-based asthma intervention: a qualitative follow-up study	Community pharmacy	Semi-structured face-to-face interviews (follow-up study)	A sample of six GPs, 10 community pharmacists and 10 patients	Community pharmacists felt that patients tend to self-medicate themselves with reliever medication and they are not interested to seek medical advice for asthma. Pharmacists had a positive assessment of the intervention and the time was not considered as a barrier for implementation. Pharmacists stated that GPs should be more involved in the intervention process.	-First study reported on the views of GPs, pharmacists and patients of such an intervention.	-Low response rate -Recruitment bias -A sample of respondents from northern and southern Tasmania only (not generalisable)	7
17 UK	Qualitative Study of Practices and Challenges of Stepping Down Asthma Medication in Primary Care Across the UK	Primary care	Qualitative methodology using a questionnaire and semi-structured interviews.	12 doctors, 9 nurses and 8 pharmacists working in primary care were interviewed.	All participants used asthma guidelines in practice, but only few of them were aware of step-down guidance. Pharmacists were familiar with SMART/MART (maintenance and reliever therapy regimens). Barriers of stepping down: Lack of awareness, fear and concerns, lack of knowledge and skills, lack of time and lack of systematic acceptance. Recommended approaches to reduce overtreatment involved education and training, improving evidence gathering, and integrating step down into routine care.	-This study presented primary care professionals' views, challenges and ideas around step down of asthma medication. -The participants were professionals with various experiences and interests.	-Recruitment bias -The researcher did not justify the reason for choosing this study design. -Not all the aspects of the research were fully discussed.	4
18 Australia	Asthma management in rural New South Wales: Perceptions of health care professionals and people with asthma	Small centre	Semi- structured interviews (Exploratory study).	Eight GPs, 10 pharmacists and 10 people with asthma	Pharmacists stated that they are responsible for dispensing, monitoring frequency of medication use, education on inhaler technique and patient support. The most important barrier for managing asthma was patients' attitude regarding their asthma and pharmacists' interventions. Other barriers to optimise asthma management were lack of time, restricted pharmacy area, lack of communication with the GPs, limited accessibility of continuing professional education and medication cost.	-This study explored the perceptions, barriers and solutions of GPs, pharmacists and patients regarding asthma management	-The analysis was done by a single researcher and it was not explained in depth. -The researcher was a pharmacist and there might be some bias in interviewing approach. -The study was done in one rural community (not generalisable).	2
8 Australia	Perspectives of pharmacists about collaborative asthma care model in primary care	Primary care	Semi-structured in-depth interviews (Qualitative inductive methods)	25 pharmacists	Barriers to optimise pharmacy-based services for asthma management were related to patient's unwillingness to seek pharmacists' assistance, time and workload limitations, factors related to health system and limited collaboration with GPs. Pharmacists suggested to develop specified channels for inter-professional communication for sharing patients' information. Also, they reported that specialised training for asthma management delivery is important with defined roles for each healthcare professionals. Barriers to implement General Practice Pharmacist (GPP) model were Funding, unwillingness to participate in this model and self-autonomy of healthcare professionals	-This study provided insight into pharmacists' perspectives about building a new role opportunity in a pragmatic model.	-Recruitment bias -The interview questions were used to retrospectively assess pharmacists' experiences (recall bias). -Pharmacists' thoughts about collaboration or implementations were based on their current practice only.	9
19 Indonesia	Pharmacists' views on the development of asthma pharmaceutical care model in Indonesia: A needs analysis study	Variety of public and private hospitals, primary health care centres, chain pharmaciesand independent community pharmacies	Focus Group Discussion (Exploratory study).	103 pharmacists	Pharmacists expressed their willingness to offer asthma services for the patients. Main barriers to service provision were lack of knowledge, lack of training, lack of supportive professional frameworks, lack of time, lack of reimbursement channels for services and lack of collaboration with doctors. Participants asked for a visionary leadership course to assist pharmacists in health services and few of them suggested that public health education is essential to understand the role of the pharmacist.	-Independent coding was used to reduce bias. -There was a detailed description on how the focus group discussion is conducted. -Quality assurance criteria were implemented. -Participants had different years of experiences and workplaces.	-The interview was not translated by professional translators. -Focus groups were conducted by different facilitators. -The study was done in one province (not generalisable).	9
20 Australia	Pharmacists' experience of asthma management in culturally and linguistically diverse (CALD) patient	Community pharmacy	semi-structured interviews using constructivist-interpretive paradigm.	32 pharmacists	Pharmacists' attitudes towards patients from CALD backgrounds were positive, though few participants emphasised concerns over the time required for counselling and a level of frustration about miscommunication. Barriers for providing care for CALD people with asthma were Language barriers, patients' health literacy, low access to translator services and cultural barriers. Strategies to overcome language/cultural barriers were hiring only bilingual staff and using audio-visual counselling aids.	-First study to explore Australian pharmacists' experience of asthma management in CALD patients with asthma. -The recruited sample provided a various level of experience expressed in the interviews. - An independent transcribing company was used to transcribe the interviews. -Quality assurance criteria were implemented.	-Convenience sampling was used (social desirability bias) -The study did not include participants as co-analyser.	9
21 Australia	Collaboration in chronic care: unpacking the relationship of pharmacists and general medical practitioners in primary care.	Primary care	semi-structured interview utilising a theoretical framework and the collection of empirical data.	18 pharmacists and 7 GPs	Pharmacists and GPs stated that they have inadequate understanding of each other's role. Pharmacists reported lack of confidence in the optimal way to reach the GP. Pharmacists emphasised on the need to improve communication with GPs (Face-to-face was the preferred method). Barriers for collaboration with GPs identified by pharmacists were financial issues, lack of time, lack of communication, GP attitudes, inaccessibility, lack of motivation to interact, low morale, *threat to GPs' role* and patient.	-This study provided a conclusion of a process for implementing collaboration in primary care based on empirical data and a theoretical framework.	-It was not clear whether the results reflected the current situation of pharmacy in Australia, or it was associated with the participants in this study only. -The relationship between researcher and participants has not been adequately considered.	5
22 Australia	Experiences of community pharmacists involved in the delivery of a specialist asthma service in Australia	Community pharmacy	focus group or semi-structured interview. A cluster randomised controlled study comparing the original service model with a less-intensive model.	32 pharmacists (25 pharmacists involved in a focus group and seven via telephone interview)	Pharmacists had positive reflection on asthma management service and there were requests for more emphasis on using spirometry to increase pharmacists' confidence. After recruiting patients for this service, patients' unwillingness to continue with the programme caused frustration for the pharmacists. Main challenges for the pharmacists were workflow, consultation location, recruitment of asthma patients, time management, and collaboration with GPs. *Reasons for miscommunication with GPs were reported as misunderstanding of the service and threat to GPs' role.*	-The combination of focus group and interview provided in-depth individual reflections with data collection. -Independent facilitator was used in this study to obtain pharmacists' feedback.	-Regardless the use of three time points for pharmacists' opinion, the study did not examine each pharmacist's experiences. -Lack of commitment of younger patients and greater involvement of older patients in the study.	6
23 Denmark	How to engage experienced medicine users at the counter for a pharmacy-based asthma inhaler service	Community pharmacy	Semi-structured interview (Exploratory study)	9 pharmacists and 3 pharmacy technicians	Participants reflected that it was easier to recruit beginners for the inhaler technique service as opposed to experienced users. Recruitment challenges were related to customers' knowledge and lack of acceptance to receive the service. The sequence of recruitment strategy was a conversation opened by a staff member asking a question which would make the customer wonder, raise interest or acknowledge regarding their treatment followed by some argument to motivate the customer to accept the service. Some staff members encouraged customers to demonstrate inhalation technique as a conclusion for the recruitment sequence (effective way).	-Validation was done by a second researcher to check the developed codes.	-Ethical approval was not obtained. -Recruitment bias -The relationship between researchers and participants has not been adequately considered. -Results attained through interviews would be different than possible results of direct observations of the recruitment process at the counter (social desirability)	6
24 Australia	A qualitative evaluation of the implementation of guidelines and a support tool for asthma management in primary care	Primary care	Focus groups (A qualitative approach underpinned epistemology)	57 stakeholders, including 19 pharmacists and asthmatic patients	Pharmacists expressed a positive attitude towards short-acting beta agonists (SABA) guidelines. Barriers recognised to the use of SABA guidelines and written asthma action plans: lack of knowledge, lack of self-competence, lack of outcome expectancy, patients' behaviour, collaborative barriers with GPs, time, workflow and guideline/resource- related barriers. Solutions for improvement: -mandating of SABA recording - using electronic resources -improving written asthma action plan ownership	-A range of stakeholders were included. -There was in depth description of the analysis process. -Using a “non-stakeholder” as a facilitator to confirm objectivity in data collection and analysis. -Multi-modal data collection was used, including field notes. -Thematic analysis was performed by two independent researchers.	-Results of the study are not generalisable. -The inadequate awareness of the resources resulted in many of the discussions being hypothetical.	9
25 Canada	How can adherence to asthma medication be enhanced? Triangulation of key asthma stakeholders' perspectives	Hospital	Focus group (Qualitative, multiple-case study)	38 asthma stakeholders, including 12 allied healthcare professionals AHPs (involving pharmacists)	AHPs believed that they can be contributors to long-term controller medication adherence by providing patients with educational support. Facilitators to long-term asthma controller medication adherence based on AHPs' perspectives were patients' knowledge, fears about an asthma exacerbation experience, acceptance of the disease, patient's attitude, accessibility to medication and patient-physician relationship. Educational intervention should be provided face-to-face, for a duration of 45 min–2 h, by a range of healthcare professionals having time to discuss many topics with patients and ready to demonstrate inhaler techniques.	-This study aided to highlight and triangulate the perspectives of patients, physicians, and AHP. -Different sexes and ages across group were sampled and supported sharing views honestly from all participants.	- Socially desirable responses were identified. -Data saturation and topic guide were not discussed. -All participants were recruited from a single urban teaching university hospital (not generalisable). -The relationship between researcher and participants has not been adequately considered. -Ethical approval from the ethics committee was not obtained.	3
26 Australia	The Role of Pharmacists in General Practice in Asthma Management: A Pilot Study	Primary care	semi-structured interviews (A pilot study).	Patients and HCPs, including five pharmacists	Pharmacists were satisfied with providing asthma care. *“One of the most satisfying things is also the asthma cycle of care. Seeing them coming back you look at their asthma, you give their education, they're coming back and they're saying they're feeling so much better”.* (Practice Pharmacist) Pharmacists believed themselves as experts in asthma care. *“If somebody comes in for asthma, everybody, even the reception, think about me”.* (Practice Pharmacist)	-Pharmacists were not asthma specialists (the data is more representative)	-Small sample size. -Recruitment of the participants was not written clearly. -Details of the interview were not mentioned. -There was no in-depth description of the analysis process. -Pharmacists were from one city in Australia (not generalisable).	1
27 England	Community pharmacy integration within the primary care pathway for people with long-term conditions: a focus group study of patients', pharmacists' and GPs' experiences and expectations	Community pharmacy	Focus group. “7Ps marketing mix” (“product”, “price”, “place”, “promotion”, “people”, “process”, “physical evidence”) was used to frame data collection and analysis.	Different stakeholder groups, including 12 pharmacists	All stakeholder groups expected that community pharmacies can offer medicines management for patients with long-term conditions (LTCs). This included ensuring appropriate medication use, educating patients, double-checking prescriptions and referring patients to GPs if required. All pharmacists estimated that patients prefer GPs and practice nurses to manage their condition and deliver clinical service. Pharmacists stated that inadequate time, workload and pharmacy managers' financial conflicts of interest, absence of private room, and the impression of a retail shop rather than a healthcare venue are barriers to implement a quality community pharmacy service. Barriers to collaboration with GPs mentioned by pharmacists are tension arising from funding conflicts and GPs' unwillingness to recognize pharmacists as healthcare providers. All stakeholder groups agreed that due to the accessibility of community pharmacies, patients preferred them over GPs for non-urgent services.	-First study to use marketing theory to understand how community pharmacy services could be better used within the primary care pathway for patients with LTCs. -This study was not restricted to a specific service. -The process of data analysis was rigorous and the interpretation was evaluated by all authors.	-Recruitment bias -Data coding was done by one researcher	8
28 Canada	A multi-stakeholder perspective on asthma care in Canada: findings from a mixed method needs assessment in the treatment and management of asthma in adults.	Community Pharmacy	Semi-structured interview and online survey. (Mixed methods approach)	43 stakeholders, including 5 pharmacists	Challenges in adult asthma care as reported by participants were implementation of guidelines into clinical practice, using spirometry, individualisation of asthma devices to patients' needs, patient adherence and self-management and clarifying roles and responsibilities of healthcare professionals. Participants reported the need to increase overall knowledge of guidelines for the asthma care. Participants reported that using spirometry and applying written care plan are not necessary for asthma management.	-Different methods of data collection were used (interviews and survey). - The recruited sample included multiple stakeholders having different years of practice.	-Participation in the study was voluntary (selection bias). -This study was done in 4 largest Canadian provinces only (not generalisable). -Areas where care was stated to be optimal were not included in the study.	7
29 U.S	Pharmaceutical Care in Chain Pharmacies: Beliefs and Attitudes of Pharmacists and Patients	Community pharmacy	Focus group	11 pharmacists and 13 patients	Pharmacists suggested that pharmaceutical care, rather than dispensing, should represent their profession's goal. None of them stated that monitoring patients' health or documenting pharmaceutical care as parts of pharmaceutical care. Pharmacists reported difficulties in communicating with physicians.; however, recent graduates were less worried about physicians' attitudes and they believed that younger physicians were more responsive to pharmacists' interventions. Barriers to pharmaceutical care implementation were time, lack of technician support, private room for counselling and uncooperative patients (especially men). Pharmacists reported that counselling on the use of inhalers or peak flow meters is time-consuming and they had to differentiate between various type of inhalers. Several pharmacists reported that they need to be trained on peak flow meters. To improve asthma counselling, pharmacists suggested additional training course to improve their knowledge, providing placebo inhalers and sample peak flow meters in pharmacies for demonstration, obtaining support from technicians.	-This study demonstrated beliefs of pharmacists and patients with asthma or COPD regarding pharmaceutical care and pharmacists' role in community pharmacy, barriers to provide pharmaceutical care and solutions to overcome these barriers.	-General characteristics of pharmacists, such as years of experience and age, were not mentioned. -Data saturation was not mentioned. -There was no in-depth description of the analysis process. -Not all the aspects of the research were discussed in discussion section. -The study was conducted in one area in U.S (not generalisable) -New areas where research is necessary and the contribution of the study to existing knowledge were not identified.	1
30 UK	Patient and Pharmacist Views of Asthma Care in the Community Pharmacy	Community pharmacy	Semi-structured interview	15 community pharmacists and 16 asthmatic patients	Pharmacists reported that inhaler counselling is not a common activity. They usually provide an explanation by a few words or refer the patient back to their doctor. Pharmacists expressed that they provide inhaler counselling in specific situations, such as first-time users, older people, children, patients using spacer devices or high dose steroid inhalers and if the GP asked the pharmacists to counsel the patient. The GP and asthma nurse were considered as the usual reference for professional asthma care. Sharing information between doctor and pharmacist was an important consideration to know what the doctor “wanted patients to know.” Pressure of work, time, patients' attitude and private room for consultation were mentioned as barriers to implement pharmacy service. Most of the pharmacists did not report an urgent need for change or to implement specialised service.	-Pharmacists were recruited from local health authority lists (to reduce bias) -The study provided insight into different pharmacists' views of asthma care (recruitment not based on asthma specialists)	-The interview details were not mentioned, such as time, place and form of data collection. -The relationship between researcher and participants has not been adequately considered. -Not all the aspects of the research were discussed in discussion section. -Limitations and implications of the study were not identified. -The study was done on a narrow geographical area (not generalisable)	2
31 Australia	Specialisation in Asthma: Current Practice and Future Roles – a Qualitative Study of Practising Community Pharmacists	Community pharmacy	Semi-structured interview (Exploratory study).	17 pharmacists (8 pharmacists that had a special interest in asthma and 9 pharmacists with no professed interest in asthma care)	Pharmacists who had an interest in asthma were more positive in providing asthma service in comparison to pharmacists in the general group. Verification of the patient's inhaler technique was not a routine activity in either group, however, demonstration of the technique was done by the pharmacists for patients using the inhaler for the first time. Pharmacists in both groups stated that counselling patients about peak flow metes and action plans is difficult. Most of the pharmacists supported specialisation within pharmacies to extend their roles. They reported that specialisation needs a trained pharmacist, private area for counselling, specialist pharmacies or pharmacists for different diseases, clinical tests for monitoring the patient, Software support, collaboration with GPs and other HCPs and financial support. Barriers to implement specialisation were time, money, GP attitude, lack of training, patient attitude, local culture and lack of coordination of professional bodies.	-The study provided insight of the level of involvement for two groups of pharmacists regarding provision of asthma care, one group that had a special interest in asthma and another group that included pharmacists with no professed interest in asthma care (diverse opinions).	-There was no in-depth description of the analysis process. -The relationship between researcher and participants has not been adequately considered. -Limitation of the study was not mentioned.	6

Bias was not assessed within or across the studies. Qualitative research is fundamentally based on the experiences of individuals', including the researchers who become part of the research.^13^

### 2.5 Data extraction and analysis

Data extraction and analysis was based on the standardised method described by Thomas and Harden^13^ and previously used by the research team.^14^ All the authors were involved in data extraction and analysis process. Relevant data (including any text or diagrams in the Introduction, Methods, Results, Discussions and Conclusions sections of the papers) was extracted from the papers as data and summarised in a table. Data were thematically analysed in stages to identify study characteristics (Stage 1 that describe the study), descriptive themes (Stage 2 which describe study findings) and analytical themes (Stage 3 which thematically synthesise data to create new findings which go beyond the findings of the original study), The characteristics of the reviewed articles are summarised in Table 2 with findings from Stage 1–3 summarised below. Thematic synthesis, alongside meta-ethnography, meta synthesis, and critical interpretive synthesis, is a suitable method for the synthesis of qualitive research in systematic reviews and is described by Thomas and Harden as providing “explicit and transparent” links between qualitative research data and findings.^13^ These links are made by including in the presentation of analytical findings, quotes and extracts of data from the original papers.

## 3 Result

### 3.1 Summary of included literature

#### 3.1.1 Stage 1 Results: Study characteristics

The majority of studies were conducted in high income countries, with most of the studies conducted in Australia (n = 10).^8^^,^^15^^,^^16^^,^^18^^,^20., 21, 22.^,^^24^^,^^26^^,^^31^ Three studies were done in UK,^17^^,^^27^^,^^30^ two studies in Canada,^25^^,^^28^ one study in Indonesia,^19^ one study in Denmark^23^ and one study in USA.^29^

Studies mostly used semi-structured interviews as a method for data collection (*n* = 11),^8^^,^^16^, 17., 18,^20^^,^^21^^,^^23^^,^^26^^,^^28^^,^^30^^,^^31^ while five studies used focus group^19^^,^^24^^,^^25^^,^^27^^,^^29^ and one study used both interviews and focus groups.^22^ Only one study investigated healthcare professionals' perspectives through the medium of drawings.^15^

Twelve studies involved different healthcare professionals or asthmatic patients with pharmacists,15., 16, 17., 18,^21^,24., 25, 26., 27., 28., 29., 30. whereas other studies included pharmacists only (*n* = 5).^8^^,^^19^^,^^20^^,^^22^^,^^31^ Only one study included pharmacists with pharmacy technicians.^23^ This suggests evidence involving the wider pharmacy team is limited.

Most of the studies were done in community pharmacies (*n* = 9),^16^^,^^20^^,^^22^^,^^23^^,^27., 28., 29., 30., 31. whereas five studies were conducted in primary care settings.^8^^,^^17^^,^^21^^,^^24^^,^^26^ Two studies were conducted in a variety of settings, including tertiary, primary and community.^15^^,^^19^ One study included one hospital only^25^ and another study was done in a small health centre.^18^

#### 3.1.2 Stage 2 Results: Descriptive themes

Major themes identified factors that influenced pharmacists' involvement in asthma care services. These were:

i) Pharmacists' perspectives of asthma treatment
ii) Pharmacists' perspectives of patients with asthma
iii) Health-system factors
iv) Inter-professional collaboration with GPs regarding asthma treatment
v) Education and training
i) **Pharmacists' perspectives of asthma treatment**

Most of the studies demonstrated that pharmacists had positive attitudes towards asthma interventions and expanding their roles in patient care.^16^^,^^19^^,^^22^^,^^26^^,^^27^^,^^29^^,^^31^ Two studies showed that pharmacists perceived themselves to be competent and experts in asthma care.^15^^,^^26^ Pharmacists explained their roles as dispensing, ensuring appropriate medication use, education on inhaler technique and patient support.^18^^,^^23^^,^^25^^,^^27^^,^^29^ However, pharmacists reported that inhaler counselling is not a common activity and they demonstrated inhaler technique only for patients using the inhaler for the first time.^30^^,^^31^ Providing asthma care plans, documentation and monitoring peak flow were considered insignificant and difficult for many pharmacists.^28^^,^^29^^,^^31^ However, pharmacists in one study believed that they could help in writing asthma care plans.^24^ Most of the pharmacists expressed a positive attitude towards asthma guidelines,^17^^,^^24^ but few pharmacists were aware of step-down guidance.^17^

ii) **Pharmacists' perspectives about patients**

The literature reported that pharmacists perceived patients did not want to engage in pharmacy-led asthma services.^15^^,^^16^^,^^18^^,^20., 21, 22., 23.^,^28., 29., 30., 31. The reason for this according to pharmacists' views was related to patients' knowledge, patients' beliefs, culture, medication cost and lack of acceptance or confidence to provide this service by pharmacists.^8^^,^^20^^,^^23^^,^^29^ Pharmacists reported young and busy adults were the most difficult patients to provide asthma care to,^28^ and other evidence^29^ reported men as the most uncooperative patients. As a result of perceived patient attitudes, pharmacists experienced fear, frustration, stress and worries about providing asthma services to patients.^15^^,^^20^^,^^22^^,^^28^ One study reported that patients' drawings encouraged the pharmacists to revisit their personal expectations of patients' illness experiences and triggered acknowledgement and empathy regarding the patient experience.^15^ Pharmacists estimated that patients prefer GPs and nurses to deliver asthma services as they are considered the usual reference for professional asthma care.^27^^,^^30^ However, limited evidence indicated, due to the accessibility of community pharmacies, patients may prefer pharmacists over GPs for non-urgent services, such as asthma reviews.^27^^,^^30^

iii) **Health-system and environmental factors**

Barriers identified in the literature that influenced pharmacists' involvement in pharmacy-led asthma services were related to the healthcare system or environment. Time limitation, workload, lack of private counselling areas and the physical environment were found in many studies as factors that limited pharmacists' abilities to get involved in providing asthma services.^8^^,^^15^^,^17., 18, 19., 20.,^22^^,^^24^^,^27., 28., 29., 30., 31. However, one study reported that time is not considered as a barrier to implement asthma intervention.^16^ Lack of reimbursement and human resources were also found as environmental or health system barriers of asthma care.^8^^,^^19^^,^^27^^,^^29^^,^^31^ Thus, financial support,^19^^,^^31^ involving extra staff, pharmacy technicians, in asthma services delivery^27^^,^^29^ and providing private area for counselling^29^^,^^31^ were identified as solutions for developing asthma care by pharmacists.

iv) **Inter-professional collaboration with GPs**

Of the reviewed literature, many studies have placed emphasis on the importance of inter-professional collaboration between healthcare professionals, especially GPs. A lack of communication was reported as a significant barrier for asthma care.^8^^,^^16^^,^^18^^,^^19^^,^^21^^,^^22^^,^^24^^,^^27^^,^^29^^,^^31^ Reasons for poor collaboration were reported as misunderstanding of the provided asthma service or each other's role, lack of pharmacists' confidence, lack of communication and difficulties in finding the optimal way to contact the GP, lack of time, GP attitude and threat to GPs' role or financial interests.^8^^,^^21^^,^^22^^,^27., 28., 29.,^31^ Collaboration with GPs was limited to serious cases only and the extent of collaboration appeared dependent on GPs' response to their recommendations.^8^ However, there was some evidence that recent graduates were less worried about physicians' attitudes and believed that younger physicians were more responsive to pharmacists' interventions.^29^ Most of the studies consistently reported the need for a shared information system to increase collaboration with GPs regarding asthma treatment.^8^^,^^21^^,^^27^^,^29., 30., 31.

v) **Education and training to deliver asthma services**

Low levels of knowledge and skills among pharmacists were reported in the literature as barriers to pharmacy-led asthma services.^8^^,^^21^^,^^27^^,^29., 30., 31. However, one study showed that there was high awareness of short-acting beta agonist (SABA) guidelines and asthma action plan card among pharmacists.^24^ Evidence suggested the necessity to educate and train pharmacists to increase their knowledge, confidence and skills in relation to asthma treatment or using spirometry and inhalers.^8^^,^^19^^,^^22^^,^^28^^,^^29^^,^^31^ Other studies mentioned special educational courses, such as a visionary leadership course in health services^19^ and health psychology to understand physical and verbal cues of patients.^23^^,^^29^ One study suggested specialist pharmacists for different diseases were needed to expand pharmacists' roles.^31^ Additionally, implementing a structured model of asthma care by clarifying the roles and responsibilities of each healthcare professionals was reported by many pharmacists to provide optimal asthma care.^8^^,^^18^^,^^28^

#### 3.1.3 Stage 3 Results: Analytical themes

Two major analytical themes that underpinned pharmacists' involvement in asthma services were identified. These were:

### 3.2 Identity

A professional identity is described as a view point of who they are in the context of their chosen profession.^32^ Although pharmacists were well educated about asthma treatment and had skills in counselling patients as stated in one study (Quote 1). The professional identity of pharmacists may influence their behaviours in practice in relation to interactions with other health care professionals or patients or self-confidence in clinical decision-making. Some data described the key aspect of pharmacists' role as dispensing the medicine to the patient (Quote 2). Other data reported that pharmacists are responsible for telling the patients about all of the information that is related to the medicine, such as side effects, uses and compliance (Quote 3,4). Thus, dispensing the medicine to the patient with the right information composes an integral part of pharmacists' professional identity.

*“If somebody comes in for asthma, everybody, even the reception, think about me”.*^26^*Quote 1 (Pharmacist)*.

*“…, we check their last supplies,.. as a backup to the GP…, just making sure they are taking their medications correctly”.*^18^*Quote 2 (Pharmacist)*.

*“First of all, we can tell them everything about the new medication: side effects, how to use it, techniques, importance of continuing to use it…making them aware of how important their medications are for them, how important it is to be compliant”.**^8^**Quote 3 (Pharmacist)*.

*“ telling patients why they take a medicine, the effects, the side effects and the outcome, and checking medication compliance”.*^29^*Quote 4 (Pharmacist)*.

Interestingly, other data stated that pharmacists reported that they would prefer to work in dispensing roles, or they were not interested in extending their role due to concerns of self-autonomy (Quote 5, 6). Additionally, other data suggested that pharmacists' identity was diminished compared to their colleagues. (Quote 7,8,9). Self-autonomy of pharmacists creates their professional identity that influences their behaviour in providing asthma services. Pharmacists did not feel that it is a part of their identity to take new roles and they want to stand in the dispensing area and do what they are told. Thus, a key part in preventing pharmacists from getting involved in asthma care services or extending their role is their identity.

*“An unexpected finding was the robust reluctance of some pharmacists to undertake new roles within the GPP model due to concerns of self-autonomy”.*^8^*Quote 5*.

*“A few of the pharmacists were nevertheless interested in extending the general scope of their work, and in having more autonomy from the prescriber in their advisory role”.*^30^*Quote 6*.

*“Some participants regarded doctors as having a ‘senior’ position in patient care, i.e. a position of professional hierarchy. These perceptions appeared to manifest as clinical hesitancy in contacting or collaborating with doctors”.*^19^*Quote 7*.

*“…. doctors still see themselves as the number one provider”.*^21^*Quote 8 (Pharmacist)*.

*“For some doctors, they look down on the pharmacist, they tell you what to do … they don't treat you equally”.*^21^*Quote 9 (Pharmacist)*.

### 3.3 Expectations

Although pharmacists had willingness to collaborate with GPs, they appeared to have limited expectations in the extent of knowledge of each other's roles (Quote10,11), have differing expectations of professional needs (Quote 12,13,14) and in their expectations of patients' needs (Quote 15,16). These variations in expectation may affect pharmacists' behaviours while providing asthma care services.

*“it*'*s a matter of both GP and pharmacist realising where they can help one another.*^18^*Quote 10 (Pharmacist)*.

*“I think asthma care could be optimized if there was more education around what the specific role of each health professional (doctor, pharmacist) was in regard to asthma care. I am not confident that all physicians are monitoring their asthma patients using spirometry/peak flow, and if pharmacists were aware of this gap in treatment they may make more of an effort to fill the gap”.*^28^*Quote 11 (Pharmacist)*.

*“the pharmacist*'*s role would be to … keep the doctor and the patient up to date on”*^21^*Quote 12 (GP)*.

*“The ideal GP would be … a good communicator and accessible”*^21^*Quote 13 (Pharmacist)*.

*“willing to view us as an equal partner”*^21^*Quote 14 (Pharmacist)*.

*with regards to providing additional information for patients (patients*' *needs),*

*“benefits from education...definitely... [as] a lot become blasé”*^21^*Quote 15 (Pharmacist)*.

*compared with GP quote,*

*“I don*'*t know whether there*'*s any extra benefit … they*'*re not listening”*^21^*Quote 16 (GP)*.

Pharmacists' behaviours appeared to be affected by healthcare professionals' expectations. If pharmacists expect not to be involved in decision-making plans of asthma care, then they will perform behaviours based on those expectations and not get involved (Quote 17).

*“My experience is that some doctors take up pharmacists' recommendations, but many doctors think that pharmacists are not able to make that sort of decision”.*^8^*Quote 17 (Pharmacist)*.

In addition, there was different expectations between pharmacists and asthmatic patients regarding asthma treatment based on expectations (Quote 18,19,20). Data indicated most pharmacists expected patients knew how to use their medication and did not want or expect pharmacists' interventions.

*“the patient already knows how to use the medications “ and that most customers “don*'*t want and don*'*t expect counseling”**^29^**Quote 18 (Pharmacist)*.

*“Several participants emphasised that there was a general lack of trust in pharmacists among patients from culturally and linguistically diverse backgrounds. The trust issue was not just regarding gender and age but also in general, regarding the profession itself”*^20^*Quote 19*.

*“Some pharmacists said it was not appropriate to delevop an asthma service, believing that the prime concern of most patients was to obtain their medicines and get away”*^30^*Quote 20*.

Patients' expectations affect pharmacists' behaviours as they assume that the patients did not want them to explain their asthma medications, so they did not perform counselling and monitoring services.

## 4 Discussion

### 4.1 Summary of findings

This review examined extant literature on pharmacists' involvement in pharmacy-led asthma services. The perspectives of pharmacists reported in the literature were positive towards asthma interventions and to expand their roles in patient care. However, the review also identified patients' attitudes and health-system factors as barriers to implementation of pharmacy-led asthma care services as well as issues with inter-professional collaboration and current levels of knowledge and skills for pharmacists, other healthcare professionals and patients with asthma who may access asthma care services. Critically, the review identified a corner stone of pharmacists' involvement in asthma care services was their own self-perceived identity, that as healthcare professionals they have knowledge and skills to provide asthma care services, but that there was an expectation that asthma care services would not be provided by pharmacists. Further exploration is needed to understand the roles and responsibilities in multidisciplinary care and provide opportunities, within practice settings, for interprofessional collaboration, training and development. Patient and public education is also essential to recognize pharmacists' roles in asthma care and thus will increase their expectation to deliver services as part of the professional identity of pharmacists.

Goffman's theory^6^ has been used to understand behaviour in everyday life and draws on perceptions of identity and expectations. Only limited literature have used this framework in relation to pharmacy; one piece of literature used this framework in pharmacy to examine one newly introduced community pharmacy service to illustrate how a policy intended to support patient medicine-taking through the extended roles of pharmacists.^33^ The other paper used this framework for interprofessional practice in hospitals and health-professional-patient relationships.^34^ A key analytical finding was that pharmacists' involvement in asthma care services were influenced by patients' and healthcare professionals' expectations. Whilst pharmacists identified as educated, well-placed healthcare professionals, pharmacists' perspectives of patient and practitioner expectations were that they would not get involved in asthma services. Drawing on Goffman's work, the actor (the pharmacist) performs a particular role based on their own interpretation of their role backstage (self-perceived identity) and their onstage role is based on the expectations of the audience (patients and healthcare professionals). However, our results indicate that pharmacists' self-perceived identity and backstage role (as well-educated healthcare professionals involved in asthma care) misaligned with the perceived expectations of patients and professionals (not involved in asthma care). This conflict may impact practice, as existing evidence^35^ reports conflict between pharmacists changing or incomplete professional self-identity formation and pharmacists' self-perceived identity has an impact on their practice. However, literature relating to the expectations of patients with asthma (i.e. the audience) and the setting of asthma care services in community pharmacy, hospital pharmacy or general practice surgeries (i.e. the stage) is limited. Evidence included in this review focused only on pharmacists' perspectives of their own role and how others perceived their role, therefore future work should seek to explore patients' and healthcare professionals' perspectives as well as the setting of asthma care services delivered by pharmacists to fully understand pharmacists roles in asthma care services.

### 4.2 Implications for practice

As pharmacists demonstrated positive attitudes towards expanding their roles in asthma, pharmacists can be ideally positioned to deliver asthma services in different settings. Pharmacists could assist asthma patients and physicians to ensure the success of asthma management plans by providing patients with suitable information and training about asthma medication, instructing correct inhalation technique and addressing patient's needs. The findings also suggest that practitioners and policy makers wishing to develop pharmacy-led asthma services should clarify the roles of pharmacists in asthma care with the public and other healthcare professionals, as well as improve training and pharmacists' perspectives of interprofessional collaboration with GPs as a critical step to resolve the mismatch of expectations and self-perceived pharmacists' identities. The findings are important for practice as they provide an opportunity for those involved in asthma care to consider the social context of pharmacists, the perceptions of role of the pharmacist in asthma care services and how patient needs and expectations may be modified to include pharmacists as members of the asthma care team. As pharmacists considered patient engagement and patient attitudes as a barrier to involvement, healthcare professionals and policy makers involved in the design and development of asthma management services should include strategies to inform expectations about pharmacists' involvement in asthma care services.

Consideration must also be given to the context of pharmacy practice in the countries where the included studies were conducted. For example, the majority of the studies were completed in Australia, where initiatives involved general practitioners collaborating with practice nurses who work within GP clinics to deliver asthma care services,^36^ suggesting physical barriers to communication between pharmacists providing asthma care in community pharmacies and the GP teams providing asthma care in general practices.^37^ Services that Australian pharmacists can provide are mainly medication focused, similarly to their counterparts in Canada, Aotearoa New Zealand, United Kingdom and Europe. Recently, there has been progression in utilising pharmacists' skills to provide asthma care in these countries. One example is that the Australian Medical Association projected a primary care model that integrated pharmacists into general practice clinics.^38^ In this model, pharmacists can undertake the task of medication review and patient education. In the United Kingdom, the National Institute of Clinical Excellence (NICE) published the results of a model where practice pharmacists supported GPs in respiratory care for patients with asthma and Chronic Obstructive Pulmonary Disease (COPD). Results showed that this model could improve patients' quality of life and adherence to therapy and reduce medication costs.^39^ This suggests in high-income countries, pharmacists provide over the counter medicines for minor ailments and in some cases are commissioned to provide medication counselling and review services for asthma patients. However, pharmacists do not necessarily have prescribing rights, which may limit their ability to improve asthma care.^40^ Whilst most of the included studies were conducted in high-income countries, pharmacists in medium to low-income countries operate within a different context, where services may be more limited to supplying medications rather than offering commissioned services. Pharmacists in these settings then may require additional steps to reach a sustainable model of asthma care delivery. Further work is needed to explore this, particularly in low and medium income countries.

### 4.3 Implications for research

This study has implications for future research as studies included largely used interview and focus group methods to explore pharmacists' involvement, with only one using visual methods (patient drawings).^15^ Future research may seek to use a wider variety of qualitative methods, such as observation and think-aloud studies, to provide deeper insights and stimulate critical reflection on current practices.^41^^,^^42^ Additionally, evidence mainly explored pharmacists' experience with doctors and other health care professionals, but few of them concentrated only on pharmacists or on pharmacists collaborating with each other. This highlights a gap in clear and specific insight regarding pharmacists' experiences of working with each other to develop asthma care services. As pharmacists' involvement in asthma care depends on professional identity and patients' and healthcare professionals' expectations, future work might focus on understanding the theoretical relationship between pharmacists' conceptions of their own identity and engagement with professional services.

Additionally, asthma care is a large concept. Although the findings suggests pharmacists have a fear that they might be inferior to medical colleagues who have a responsibility in diagnostics and therapeutics (e.g. what kind of medication with what kind of dosing, as well as advice on weight control, exercise and smoking cessation) or nursing colleagues who provide practical guidance on taking medicines (e,g, how to remember to take the medicine, how to recognize and to remember how to increase medication during exacerbation, inhalation technique and advising non-pharmacological interventions for asthma), further research might identify a new role for pharmacists that is uniquely their own role, that does not try to replicate or replace the role of other healthcare professionals. Thus, all these health-care professionals would have their own role in asthma care and together they could support patients in better asthma care. Further research may also endeavour to quantify the role and experiences of pharmacists' providing asthma care services. For example, our findings indicate young and busy adults may be challenging to provide services to, however the nature of qualitative research does not quantify this and raises other questions (e.g. How young? How busy?). Therefore, further work is needed to answer these questions.

### 4.4 Limitations of this review

The findings of this review may be limited to high-income countries as most studies were carried out in Australia (*n* = 10) and other high-income countries. Of the 18 studies reviewed, only one study was conducted in low to middle income countries (Indonesia). Existing findings may not be applicable to other countries with different organisational and administrative health care systems, however, findings may be transferable to similar contexts.

## 5 Conclusions

This review aimed to explore factors that influence pharmacists' involvement in pharmacy-led asthma care services. It demonstrates pharmacists self-identified as capable of delivering asthma services, equipped with appropriate knowledge and skills, but the expectations of patients and other healthcare professionals prohibited their involvement in delivering asthma care services. Current evidence highlights pharmacists' perspectives of asthma treatment and patients with asthma as well as the health system factors, interprofessional collaboration with general practitioners and education and training influence involvement in pharmacy-led asthma care services. This review also identified a need for further research to better understand the experiences of and barriers to pharmacists' involvement in asthma care services in low- and middle-income countries.

## Declaration of Competing Interest

The authors do not have any conflict of interest or financial interest to disclose.
